# Evaluation of Aquaporin-Incorporated Forward Osmosis Membrane and Biofilm Carrier Materials in a Novel Osmotic Membrane Bioreactor for Low-Temperature Rural Sewage Treatment

**DOI:** 10.3390/ma19163395

**Published:** 2026-08-10

**Authors:** Li Qi, Jie Wang, Xinbo Zhang, Hui Jia, Yun Wu, Haitao Wen

**Affiliations:** 1 State Key Laboratory of Advanced Separation Membrane Materials, Tiangong University, Tianjin 300387, China; 13821460416@163.com (L.Q.); ajiahui@163.com (H.J.); wucloud@163.com (Y.W.); 2 School of Material Science and Engineering, Tiangong University, Tianjin 300387, China; 3 School of Environmental and Municipal Engineering, Tianjin Chengjian University, Tianjin 300384, China; zxbcj2006@126.com (X.Z.); 18622041916@126.com (H.W.); 4 Hebei Industrial Technology Research Institute of Membranes, Cangzhou Institute of Tiangong University, Cangzhou 061000, China; 5 School of Environmental Science and Engineering, Tiangong University, Tianjin 300387, China

**Keywords:** osmotic membrane bioreactor, fixed biofilm, rural sewage treatment, low C/N ratio, low temperature, salinity build-up, simultaneous nitrification–denitrification

## Abstract

**Highlights:**

- A novel OMBR integrated with fixed -biofilm was demonstrated.
- Aquaporin-incorporated FO membrane exhibited low reverse salt flux.
- Simultaneous nitrification–denitrification process has a certain possibility in BF-OMBR.
- Fixed-biofilm carrier material enhanced nitrification at 10 °C.

**Abstract:**

Transforming traditional membrane bioreactors (MBRs) into forward osmosis membrane bioreactors (OMBRs) is a highly challenging yet promising technological upgrade. Although both combine biological treatment and membrane separation, their core driving forces and operating mechanisms are completely different (an MBR is pressure-driven, while an OMBR is osmosis-driven). In this paper, a novel OMBR with an integrated fixed biofilm (BF-OMBR) was tested for the treatment of synthetic rural wastewater using fertilizer potassium chloride (KCl) as the draw solution (DS) and a commercial aquaporin Inside^TM^ forward osmosis (FO) membrane. A bench-scale investigation was conducted to compare the BF-OMBR with traditional OMBRs and MBRs. The experimental data suggested that the reactor with aquaporin membranes contributed to a higher water flux than traditional TFC membranes, while immobilized biofilms improved the total nitrogen removal rate compared to normal OMBRs. The integration of these two technologies in the BF-OMBR system appears to leverage these individual benefits. Its TOC and ammonia nitrogen removal efficiencies were also better than those of the other two bioreactors. Meanwhile, the BF-OMBR successfully controlled the salinity build-up to a level not exceeding 2.5 mS/cm over 90 days of operation. This novel osmotic bioreactor may represent a possible alternative approach to overcoming the challenges of low-temperature and low-C/N-ratio rural sewage treatment.

## 1. Introduction

Rural domestic wastewater increasingly exhibits a low carbon-to-nitrogen (C/N) ratio, particularly in regions where sewage is diluted by rainwater or mixed with wastewater from the livestock, poultry, and small-scale village industries [1,2]. This wastewater is typically characterized by low COD concentrations and relatively high nitrogen contents. Because drainage and collection facilities in many rural areas remain insufficient, untreated or partially treated wastewater is often discharged directly into natural water bodies. Such a discharge can accelerate eutrophication, increase the aquatic toxicity, and pose risks to environmental and public health [3].

The current treatment of these low-C/N-ratio rural domestic wastewaters have focused on the membrane bioreactor (MBR) process. In fact, many MBRs in rural domestic sewage treatment show an unsatisfactory efficiency when the main influent has a lower C/N ratio during the winter period, especially in northern China (e.g., 13 established towns in the Jinghai district of Tianjin City, China). This is because the microbial activity in MBRs is greatly inhibited in low-temperature environments, and the aerobic sludge even disintegrates under continuous decreases in temperature, resulting in a sharp decline in treatment efficiency [4]. In addition, due to the frequent use of strong alkaline or oxidizing agents such as sodium hypochlorite (NaClO) and sodium hydroxide (NaOH) for cleaning MBR systems, these highly corrosive agents inevitably accelerate the structural degradation and corrosion of stainless steel membrane frames and metal pipelines [5]. Hence, there is an urgent need to effectively investigate some alternative ways to complement the existing MBR facilities under such an emergency.

Forward osmosis (FO) is a relatively new osmotic-driven membrane separation technology developed in recent years. Compared to pressure-driven membrane separation processes such as microfiltration (MF), ultrafiltration (UF), and reverse osmosis (RO), the FO process offers many unique advantages, including a low energy consumption, high pollutant rejection, simple process operation, and lower membrane fouling propensity. These features demonstrate good application prospects in many fields, especially in seawater desalination, landfill leachate treatment, and wastewater treatment [6,7,8]. Furthermore, the combination of FO and activated sludge processes into an osmotic membrane bioreactor (OMBR) has attracted more interest in wastewater treatment and regeneration [9,10]. Moreover, for some rural applications, choosing fertilizer solutions as DS (FDFO) directly for irrigation or greenhouse hydroponic applications without any other recovery process makes the OMBR treatment more economical and suitable for wastewater treatment [11,12]. Although Blandin et al. (2018) discussed a retrofit from existing MBRs to submerged OMBRs using a commercial TFC membrane and obtained a high rejection of trace organic matters, the transformation from MBRs to OMBRs has not paid attention yet to treating the low-temperature and low-C/N-ratio sewage prevalent in rural areas [13]. Meanwhile, research on the salinity accumulation in the modified OMBR is relatively limited. Salt not only damages the microorganisms and sludge in the system, but also poses a risk of equipment corrosion [14]. Furthermore, the investigation of total nitrogen (TN) removal in retrofitting MBR to OMBR is still inadequate. An investigation of directly retrofitting MBR to OMBR, especially in combination with some other process (e.g., immobilized biofilm carrier, or sponge-based moving bed), might be important and valuable, and urgently needed in the future.

In this study, a lab-scale experiment was applied to establish a novel OMBR system combined with an AQP-FO membrane and immobilized biofilm, with the expectation that the existing MBR system can be supplemented by this novel OMBR system as an alternative to ensure the safety of the effluent discharge in winter or other extreme conditions. We explore this novel OMBR’s performance in treating low-temperature and low-C/N-ratio wastewater compared with MBR systems and other normal OMBR systems, and investigate the effect of water flux, ammonia nitrogen and TOC removal, fouling and backwashing, and mixed liquor salinity build-up in a relative long period, with the aim to use OMBR to replace MBR as the emergency response to guarantee the standard of the effluent quality under low influent C/N ratios in winter.

## 2. Materials and Methods

### 2.1. The MBR, OMBR, and BF-OMBR System

**Figure 1:**
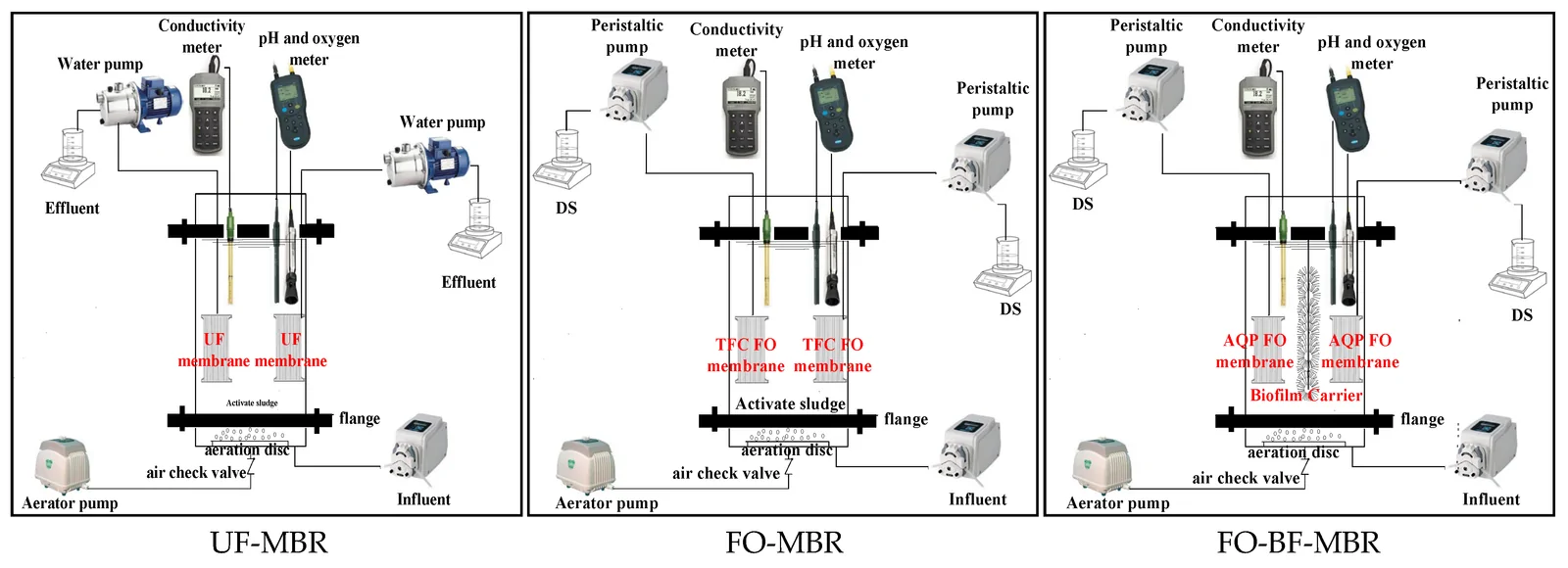
Schematic diagrams of the MBR, OMBR, and BF-OMBR systems.

The experiments were conducted during the course of 90 days (March–June 2025). The lab-scale MBR system (reactor-1) used in the experiment was evaluated in a submerged mode setup and had an effective volume of 50.2 L. The corresponding normal OMBR (reactor-2) and BF-OMBR (reactor-3) were also prepared according to this size (Figure 1). All three reactors were placed in a rectangular pool; low-temperature circumstance in the pool was provided by low-temperature circulator (HX-08, BILON, Shanghai, China), and all the reactor’s water temperature were controlled at 10 ± 1.5 °C on average.

The aerobic activated sludge collected from Xianyang Road Sewage Treatment Plant (39°06′14.78″ N 117°04′49.66″ E, Tianjin, China) was used as seed-activated sludge for all the reactors and the concentration of the seed sludge was 7 ± 1 gMLVSS/L initially. Air was continuously pumped independently into these reactors under the UF or FO membranes at 1 L/min during system operation. The hydraulic retention time (HRT) and the sludge retention time (SRT) of reactors were chosen in 6.6 h and 21 days, respectively.

The combined-type double-ring filler (Table 1) in reactor-3 was obtained from CNCLEAR (Tianjin, China) and a series of double circle plastic rings was used as the skeleton, comprising aldehyde fiber bundle, cannula, and center rope. Due to the volume limitation of the reactor, such seven rings were added in the reactor and the spacing of each ring is about 5 cm.

One peristaltic pump (BT50-1J-JY10, Longer, Baoding, China) was used to send feed synthetic wastewater into corresponded bioreactors. Another peristaltic pump (WT600-2J-YZ1515X, Longer, Baoding, China) was used to circulate the DS to the immersed membrane cell in normal OMBR and BF-OMBR at the same flow rate of 0.75 L/min, corresponding to a cross-flow velocity of 5.2 cm/s.

### 2.2. Synthetic Wastewater

The synthetic wastewater used in this study consisted of glucose, peptone, potassium dihydrogen phosphate, calcium chloride, magnesium sulphate, ferrous sulfate, sodium acetate, and urea: this information is provided in Table 2. All chemicals in this research were of analytical reagent grade and purchased from Aladdin (Shanghai, China). These chemicals are used to simulate wastewater with low C/N ratio (about 1:1.2~1.3) in this study. The feed solution (FS) influent to these reactors was the same and prepared every three days.

### 2.3. UF and FO Membranes

A flat-sheet ultrafiltration membrane was provided by Rising Sun Corporation (Beijing, China) used in this study, with a molecular weight cutoff (MWCO) of 50,000 Da. The traditional TFC FO membrane and AQP FO membrane (HTI, Albany, OR, USA) used in this study also has a flat-sheet shape, obtained from Tiangong University State Key Laboratory of Separation Membrane and Membrane Process and Botong Separation Membrane Technology (Beijing, China), respectively. The effective membrane area, whether UF or FO, was all the same, 0.014 m^2^. The membrane coupon was rinsed with anhydrous ethanol and then immersed in DI water for 0.5 h before all experiments. The active layer facing wastewater (AF-DS) orientation was adopted in two OMBRs. The FO membrane module was made of PE plastic and had a 7 × 2 × 1 cm draw solution flow chamber. The basic information of two membranes and test conditions of experiment were listed in Table 3 and Table 4.

### 2.4. Scanning Electron Microscopy

Membrane surface can cross-sectional morphology of the AQP FO membranes was characterized with GEMINISEM 500 (Oberkochen, Germany); field emission scanning electron microscope (FESEM) was used. Firstly, the membrane was cut into a rectangle and immediately immersed in liquid nitrogen, and, after determining the precise cross-sectional structure of the membrane, the membrane was quenched from the middle to obtain a complete and good cross-sectional structure. Then, the film sample after vacuum-drying is fixed on the vertical surface of the sample table with conductive adhesive to ensure that the section is upward, and the sample surface is sprayed by ion-sputtering instrument with platinum nanoparticles. Set the microscope acceleration voltage to 10 kV.

### 2.5. Draw Solutions

The DS used in this study was fertilizer KCl (analytical-grade) dissolved in deionized (DI) water (Milli-Qgard AZ MingChe^®^ Integral, Merck Millipore, Shanghai, China). The concentration of the DS was 1M. The DS was refreshed every day to recover the initial driving force of the system. The diluted DS was used to analyze TOC, NH_3_-N, and TN concentrations, and then stored in a plastic tank per day for greenhouse hydroponics.

### 2.6. Flux

The permeate flux of OMBR was determined by measuring the change in DS weight using a scale (ME2002T, Mettler-Toledo, Shanghai, China) through the Serial Debug Assistant, a computer software (Version 3.8.6). The DS volumetric change was auto-monitored at 5 min intervals. Record the data of scale at 4, 8, 12, 16, and 20 h after the start of the experiment every day for water flux calculation. The water flux (L·m^−2^h^−1^) was determined by the following equation, Equation (1):

J_W_ = ΔV/ρ·A·Δt (1)

where J_W_ is the water flux of FO (l·m^−2^h^−1^), ΔV is the volume of the water permeated from the reactor to the DS (l), ρ is the density of water (kg/L), A is the effective membrane surface area (m^2^), and Δt is the duration of the experiment (h).

### 2.7. Membrane Cleaning

The UF membrane cleaning followed the article of Holloway et al. (2015) [15]. A cleaning protocol of FO membrane consisted of two steps: (1) rinsing with DI water for 0.5 h firstly, and (2) a following backwashing process with DI water as DS and 1M DS as FS was conducted for 2 h, and was applied after the experiment finished. Before and after cleaning, the permeation flux of the membrane was determined and assessed in Section 3.1. This allowed us to evaluate (1) membrane fouling, (2) the efficiency of the cleaning protocol, and (3) potential loss of membrane integrity [16]. The recovery flux (F_rr_) was determined by the following equation, Equation (2), which reflects the proportion of water flux that can be restored after membrane cleaning expressed as a percentage:

F_rr_ = F_c_/F_i_ × 100% (2)

where F_rr_ is the water flux recovery rate, F_i_ is the initial water flux, and F_c_ is the flux after cleaning.

### 2.8. Salinity

The salinity in the bioreactors consisted of the salts in synthetic wastewater and reverse salt flux (RSF) from DS during the experiments. RSF, the diffusion amount of solute in DS that reversely infiltrates into FS through the FO membrane, was inevitable in the FO process. A conductivity meter (HI98197, HANNA, Woonsocket, RI, USA) was used to measure the conductivity and the temperature of FS and the mixed liquor in OMBR. It was determined by the following equation, Equation (3):

(3) $$J_{S}=\frac{\left(C_{f}V_{f}\right)-(C_{0}V_{0})}{t\times A_{m}}$$

where C_f_ and V_f_ represent the final FS concentration and final FS volume, respectively; C_0_ and V_0_ are the initial FS concentration and initial FS volume, respectively; A_m_ is the effective membrane surface area (m^2^); and t is the duration of the experiment (h).

### 2.9. pH, TOC, Ammonia Nitrogen, Nitrite, SVI, and DO

The pH value and dissolved oxygen (DO) was measured by a Dual-Input Digital Multi-Parameter Meter (HQ40d, HACH, Loveland, CO, USA). The total organic carbon (TOC) was conducted by a TOC analyzer (TOC-V_CPH_, Shimadzu, Kyoto, Japan). The chemical oxygen demand (COD) was measured by COD analyzer (DR6000, HACH, Loveland, CO, USA). The ammonia nitrogen and nitrite concentrations were analyzed by an Ion Chromatograph (ICS-1500, THERMO, Waltham, MA, USA) every day and per 10 days, respectively. All the samples above were passed through a microfiber filter (0.45 μm) before test.

The SVI was measured weekly from mixed liquor in three reactors according to the previously described method [17]. The DO of inner biofilm carrier was measured through Unisense Micro Profiling System with a 50 μm-tip Clark-type oxygen microsensor (OX-50, Unisense, Aarhus, Denmark).

## 3. Results

### 3.1. Membranes Characterization

**Figure 2:**
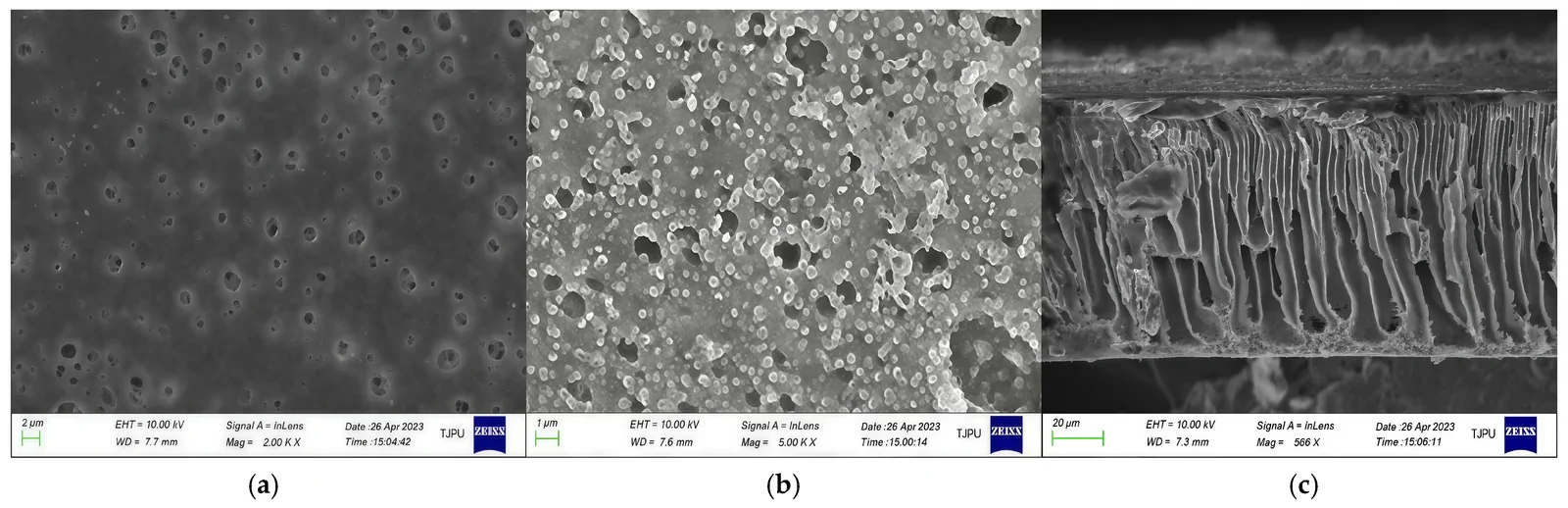
SEM images of AQP FO membrane: (**a**) bottom surface, (**b**) top surface, and (**c**) cross-section.

The bottom surface of the AQP membrane support (Figure 2a) exhibited a porous structure with pore sizes ranging from 100 to 2000 nm. For the membrane selective layer formed by interfacial polymerization, a typical rock-like polyamide morphology can be observed. The membrane cross-section shows a vertically aligned porous structure, which usually implies a higher water flux.

### 3.2. Water Flux

**Figure 3:**
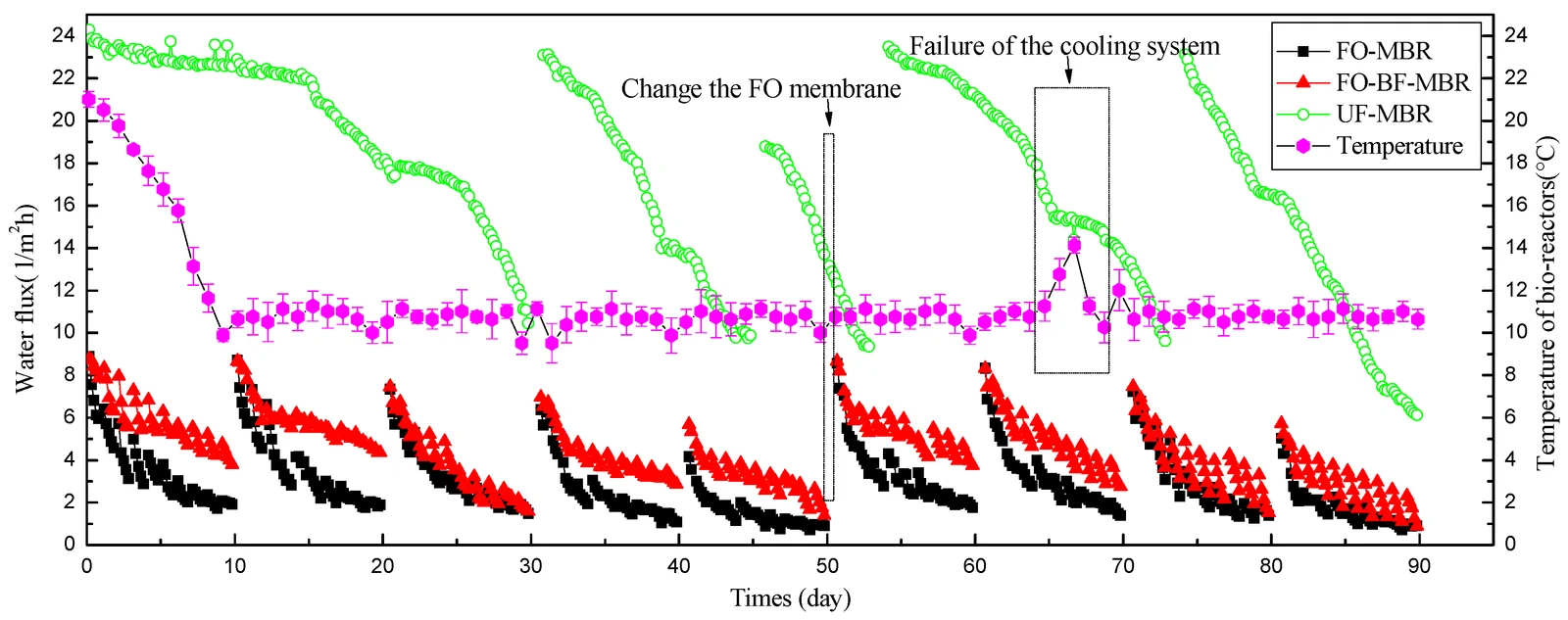
Water flux of different bioreactors during experiment.

The water flux of the membrane bioreactor is not only the most intuitive parameter for measuring the water production efficiency of the system, but also the core hub for the engineering design, operating cost control, and membrane fouling management. It should also be noted that, due to fundamental differences in the driving mechanisms, the water flux in MBR and OMBR represents different physical meanings and should not be used as an absolute equivalent indicator of the inherent permeability of membrane materials. Figure 3 shows the water flux of the different bioreactors during the 90-day experiment. The MBR system exhibited the highest water flux among the three, ranging from approximately 10 to 24 LMH. This is likely because the MBR is a pressure-driven membrane process; the membrane can exert its permeability under external forces when a certain transmembrane flow rate is set [16]. When the experiment started, the effluent flux of MBR declined slowly, while its water temperature decreased gradually from 18 °C to 10 °C. When the water temperature has been controlled at about 10 °C through the heat exchange process caused by circulating cooling water, 5 days later, the MBR flux curve in Figure 3 showed a sharply drop down. These might be attributed to the UF membrane fouling layer caused by fine particles, colloids, or polymers released from the activated sludge due to low C/N and low temperatures, which blocked the permeation of the UF membrane, leading to a high tendency of membrane fouling [17]. Moreover, these results indicated that either low C/N or low temperature resulted in the activated sludge disintegration and released too much extracellular polymeric substances (EPSs) [18]. However, this disintegration process was relatively slow and was not triggered until the temperature dropped to nearly 10 °C, likely becoming aggravated as the temperature persisted around 10 °C. This probably explains the sudden decrease in water flux five days after reaching 10 °C.

It can also be seen from the Figure 3 that BF-OMBR with an aquaporin-based FO membrane is superior to OMBR with a conventional TFC membrane in terms of water flux. This is because the traditional TFC membrane has a higher resistance when the polyamide layer is dense; the aquaporin membrane embeds the natural aquaporin in biological cells into the polymer matrix, thus forming a highly hydrophilic exclusive channel, which greatly reduces the transmembrane transport resistance of water molecules. The same conclusion can be drawn from the water contact angles of the two membranes in Table 3—that the water contact angle of the AQP FO membrane is significantly lower than that of the TFC FO membrane—which means that the AQP FO membrane is more hydrophilic and can obtain a better water flux. Apparently, the observed flux differences in this study mainly reflect the comprehensive mass transfer performance of the two systems under specific operating conditions, rather than a direct comparison of the water permeability coefficient of the membrane material itself. To compare the inherent properties of the membranes, independent standard tests (such as using pure water in PRO/FO mode) must be conducted to remove the interference from system factors.

The washing operation of the original UF membrane was conducted on day 30 and 45 when the water flux was at a relevant level that is much too low, and presented an unacceptable water production level compare to the initial one. Though the recovery water flux of the just-washed UF membrane had performed well compared to the original UF membrane, the water flux came down rapidly in the next few days and showed a shorter period than the first time of use. The same pattern also appeared in the second cleaning of the UF membrane. Thus, on day 53, we used a new UF membrane to replace the original UF membrane; for this reason, its water flux was dropped to about 60% (from 23.88 to 9.56 L/m^2^·h on average). And, on day 73 of the experiment, the washing operation of the second UF membrane was conducted a second time. But the water flux of the washed UF membrane still went down at an accelerating rate owing to the fact that the membrane pores were clogged by the aggravation of the membrane fouling. This observation was in accordance with the previous study [19]. When the experiment reached 65 days, the cooling-system malfunctioned and stopped for more than 6 days. We tried to add ice cubes produced by the ice maker every day according to the temperature measured to keep the water temperature steady in the three bioreactors. But there was still a temperature fluctuation on such days. Fortunately, these temperature fluctuations had little impact on the mode of any membrane’s water flux.

As for FO bioreactors, in accordance with the previous studies, the lower permeate flux of the FO process has always been the main disadvantage for this membrane process, which was driven by osmotic pressure, and not by some other outer pressures. Therefore, the water flux of the different retrofitted OMBR required much attention even if it was designed to be used in winter, and the low-C/N wastewater currents showed non-continuous flow. According to Figure 3, we could also see the water flux of OMBR was gradually decreased as the experiment proceeds regardless of whether the biofilm was adopted. The reasons for this flux decline might be ascribed to the following aspects: (1) the dilution of the DS weakened the osmosis-driven force, (2) membrane fouling restrained the permeability, (3) internal concentration polarization (ICP) interfered with the filtration, and (4) the RSF retarded the FO diffusion [20,21]. Figure 3 also showed evidence that the recovery of the water flux in two OMBRs due to the daily replacement of the DS also presents a decline trend; this result is mainly attributed to the aforementioned reasons (2) and/or (3). This is probably because a water flux decline seemed to be inevitable in membrane water treatment technology. Therefore, the various investigations on FO technology, such as the preparation and modification of the membrane and the development of a novel DS, and so on, did not thoroughly solve this problem but only mitigated it.

Many preliminary investigations have found that one of the advantages of the FO process was its lower fouling tendency, and the fouling layer presence showed more softness than other pressure-driven membrane processes such as MF and UF [22]. But, in this study, as described in Figure 3, after a period of use, the water production of two FO membranes also decreased rapidly and needed a membrane clean. The first FO membrane washing occurred on the tenth day after the start of the experiment when the average water flux from the first day to the ninth day dropped to 28.31% and 50.46% in OMBR and BF-OMBR, respectively. Since then, the washing protocol was conducted every 10 days from the start of the experiments to the end. It is important to note that the initial FO membrane was also replaced by a new one nearly 50 days after the experiment began due to its lower appearance of water flux (lower than 1 L/m^2^·h in OMBR). Hence, there were seven times the number of membrane clean operations altogether in the 90-day experiment.

**Figure 4:**
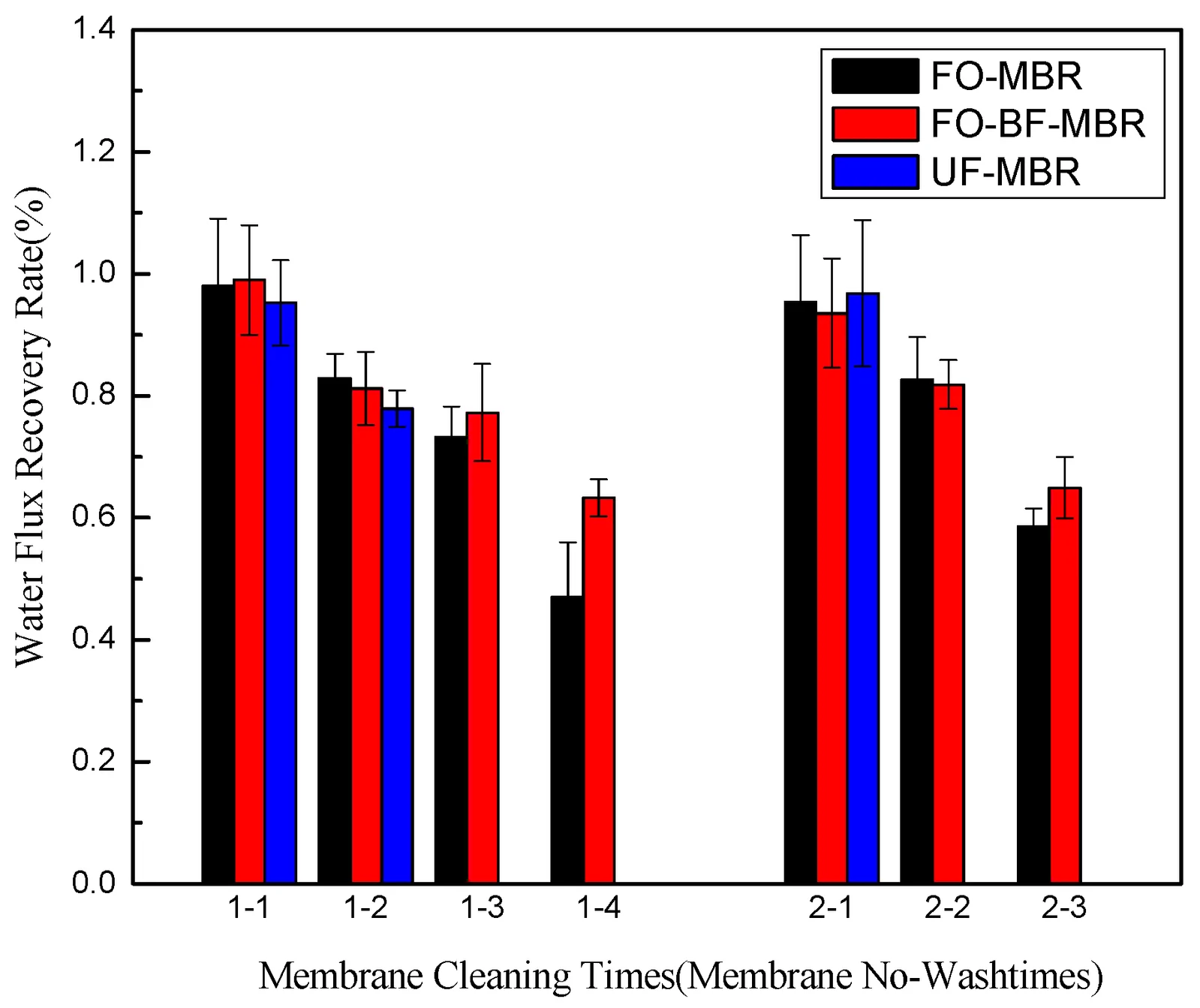
Water flux recovery rates of different bioreactors.

The water flux recovery results for the UF and FO membranes are shown in Figure 4. The analysis data are presented as the mean ± standard deviation (SD) of three independent experiments (*n* = 3). It is evident that all water flux recovery rates reached almost >95% after the first washing operation, regardless of the membrane type. Even so, the FO membrane in the BF-OMBR had the best water recovery rate compared to that in the conventional OMBR and the MBR. However, the recovery rate of all membranes showed a significant decline after the second cleaning operation. Furthermore, the recovery rate decreased to 47% and 63.3% at the fourth cleaning operation for the conventional OMBR and BF-OMBR, respectively. This indicated that FO membrane washing operations are not unlimited, as the recovered flux could not be restored to the initial permeate flux level after three or four cleanings. The same conclusion can be drawn from Figure 3. The reasons are likely due to membrane biofoulings that had adhered and clogged the membrane micropores, causing the washing protocol to be unable to achieve satisfactory results, even with an increased number of cleanings. More cleaning protocols should be developed to solve this problem in the future [23].

Comparing the behavior of the UF and FO membranes under the same experimental conditions, we know that the FO membrane’s water flux was far lower than that of the UF membrane’s. Although they had the same frequency of membrane replacement (once), the UF membrane required only three washings, whereas the FO membrane required seven washings throughout the entire experiment.

### 3.3. Salinity Build-Up

**Figure 5:**
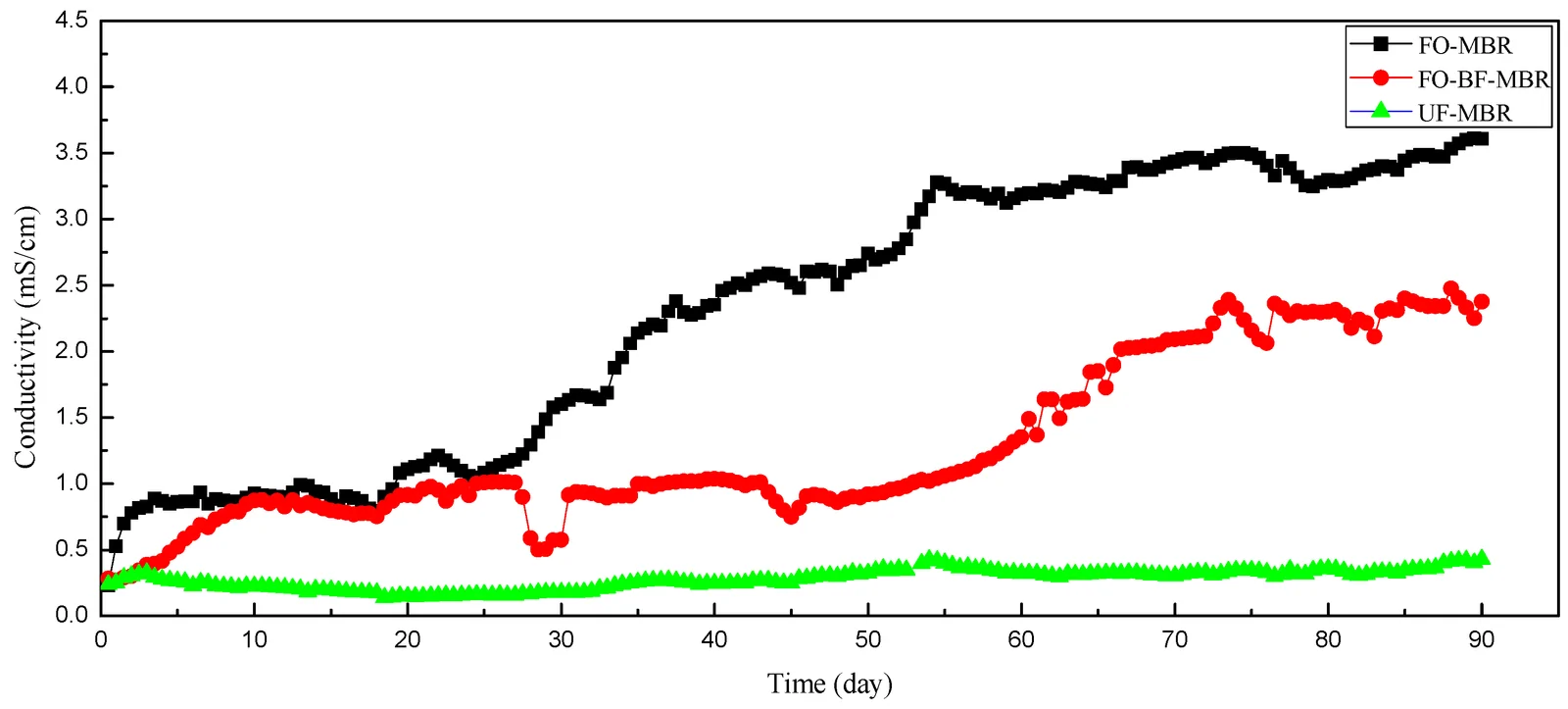
Salinity build-up in three bioreactors during the experiment.

Based on conductivity monitoring, the apparent salt accumulation trend within the bioreactor is consistent with the theoretical expectation of draw solute reverse diffusion. The salinity build-up in the reactor is a significant drawback in the OMBR process, and mainly comes from the reverse solute flux (RSF) of the DS rather than the influent water, for it threatens to accelerate the release of extracellular polymeric substance (EPS), restrain the biological activities of activated sludge, and, in extreme cases, disintegrate the sludge flocs of the biofilm, and, finally, inhibit the biodegradation of contaminants [24,25,26]. The conductivities of mixed liquor in different bioreactors are shown in Figure 5.

As shown, the three bioreactors exhibited different salinity patterns, with the conventional OMBR showing the highest salinity level and the MBR the lowest. This is likely because the UF membrane in the MBR cannot reject salts; for this reason, the MBR consistently maintained the lowest salinity level during the experiment. For the BF-OMBR, its lower salinity level compared to the conventional OMBR may be because of the following: (1) It effectively suppresses the increase in salinity through intermittent sludge discharge. The sludge discharge process also discharges the salt concentration from the mixture based on the designed SRT. In theory, more salt could be discharged from the OMBR with a shortened SRT, thereby alleviating the salinity accumulation [27]. (2) The selectivity of the aquaporin FO membrane is better than that of the traditional TFC FO membrane, and its internal channels only allow water molecules to pass through, which can effectively reject salt ions and other solutes, which leads to the result that BF-OMBR is better than the traditional OMBR in RSF control [28].

We could also see in Figure 5 that all the conductivities constantly stayed in the range of 0.2~3.3 mS/cm during the whole experiment time no matter which bioreactors were used. This salinity level was within an acceptable range; therefore, the activated sludge in these bioreactors could effectively adapt to the wastewater and withstand such levels of salinity stress [29]. Previous studies already indicate the integration of the MF/UF membrane could decrease the salinity build-up in the bioreactor effectively [25,30]. Meanwhile, some studies showed the different salinity limitations on biological treatments in the traditional OMBR, such as 34 mS/cm, 35 mS/cm, and 25 mS/cm, would break down the biodegradation process beyond this limitation [20,30,31]. In our studies, the salinity build-up was still under a low level (<3.5 mS/cm) mainly because of the sludge discharging; another reason might be due to the fact that nitrate generation partially inhibits the RSF through the AQP membrane to the FO-based bioreactors. These could be found in the nutrient removal in Section 3.4. Hence, the disintegration of activated sludge occurred not owing to the salinity build-up but the lower temperature and lower C/N of wastewater. These levels of salinity could not affect the quality of effluence even in the traditional OMBR.

### 3.4. Removal of TOC and Ammonia Nitrogen

**Figure 6:**
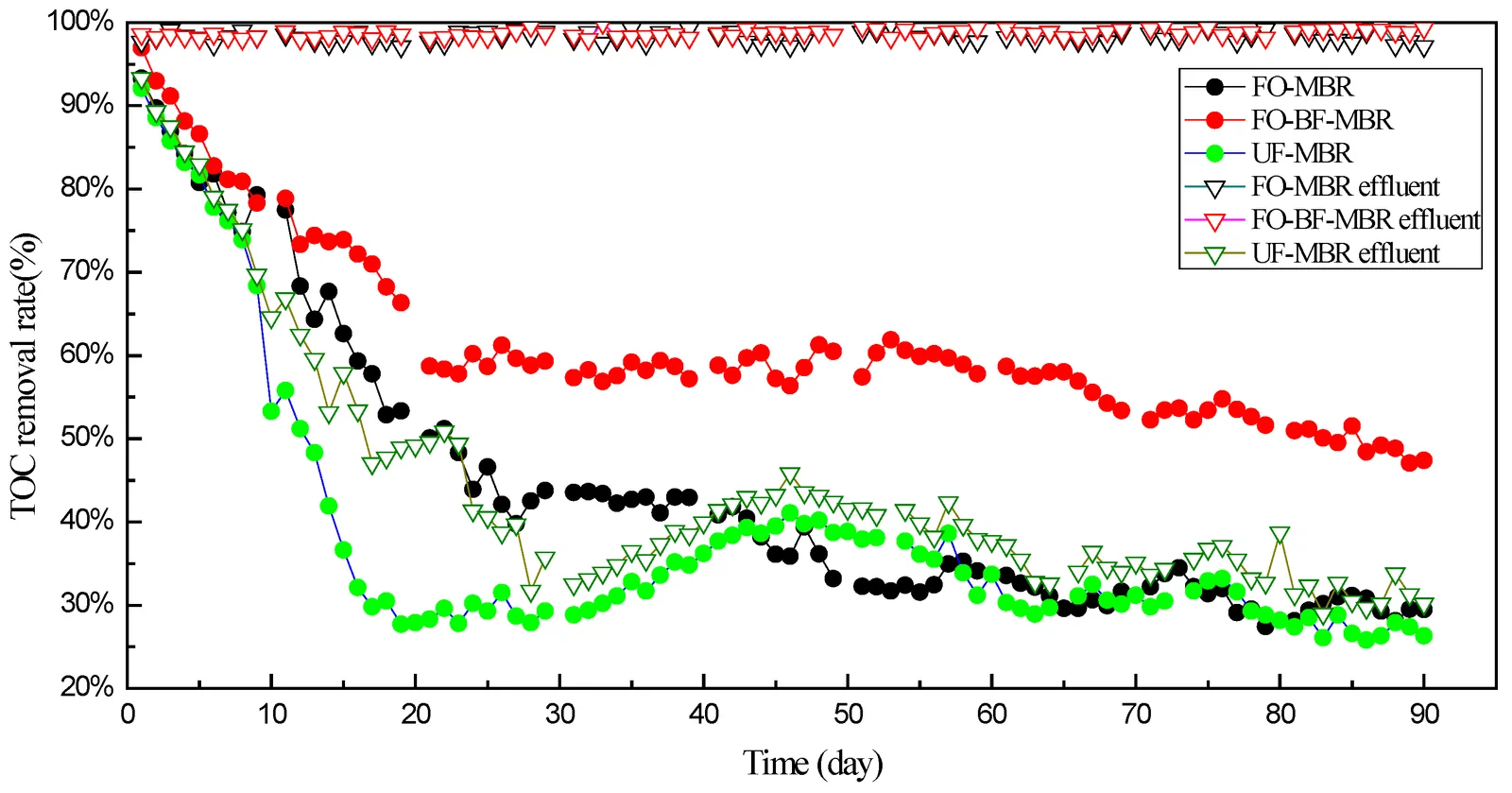
TOC removal efficiencies of the different bioreactors.

The removal of TOC in three bioreactors under a low temperature and low C/N ratios can be seen from Figure 6.

Apparently, although the TOC concentration of the mixed liquor varied in different bioreactors, the effluent removal rate of TOC in the DS of OMBR and BF-OMBR was all at the highest level not less than 97.1% and 98.2%, respectively. The TOC removal rate of the FO membrane was all above 97%, which indicated the FO membrane performed a better rejection of organic contaminants than the UF membrane despite the lower temperature and low C/N ratio circumstance; these results coincide with those of the previous studies [25,32]. Moreover, the TOC removal rate of the mixed liquor in BF-OMBR was also at a relatively high degree during the experiments compared to OMBR and MBR; these observations indicated the high TOC removal rate might be ascribed to the higher concentration of MLSS in BF-OMBR, and the long-term good performance of TOC rejection relied on the FO membrane and the biodegradation of biofilm as well. It is also easier to determine what the contribution of the removal rate was from the FO membrane’s rejection or the biodegradation of completely mixed activated sludge or biofilms. We could compare its difference of the TOC removal rate in the effluent and the mixture in the same bioreactors. On the contrary, the TOC removal of UF shows a lower ability compare to FO; this may be attributed to the characteristics of the membrane, especially in smaller membrane pore sizes in FO than UF [33].

As shown in Figure 6, all of the TOC removal rate curves showed a rapid downward trend at 20 days after the start of experiment. According to Section 3.2, it took 10 days for the mixed liquor temperature to drop from 18 °C to 10 °C; the activated sludge was subjected to a large impact of the temperature change violently in a short time. Furthermore, the low-C/N-ratio synthetic wastewater influent continuously, at the same time, also seriously affected the biological activity of the biomass. As a result, the biodegradability of the wastewater deteriorated, which made the TOC removal rate in the mixed solution decrease dramatically [34]. With the gradual increase in the experimental duration, the TOC removal rate was shown to be fluctuating in a certain range; these results might probably be ascribed to the adaptability of the activated sludge to the circumstance of the low-temperature and low-C/N-ratio influent. The biodegradability of the activated sludge was partially recovered so the TOC removal rate stabilized relatively and even showed some improvement in the days between the 30th and 45th day. However, the activated sludge under low-temperature circumstances began to disintegrate gradually, while the low-C/N-ratio synthetic wastewater influent continuously caused the decrease in the biodegradability of wastewater to be inevitable. Thus, the TOC removal rate showed a slow downward trend after 50 days from the beginning of the experiment. It was worth mentioning that the BF-OMBR showed the best presence in the TOC removal rate during the experiment: it not only relied on the perfect rejection of the FO membrane, but is also attributed to the attached-growth biofilm degradation The most likely reason was the fixed biofilm could offer better resistance to low-temperature and low-C/N-ratio influent impacts due to the attachment of the activated sludge to the framework of the carrier rather than being scattered in the bioreactor [35].

**Figure 7:**
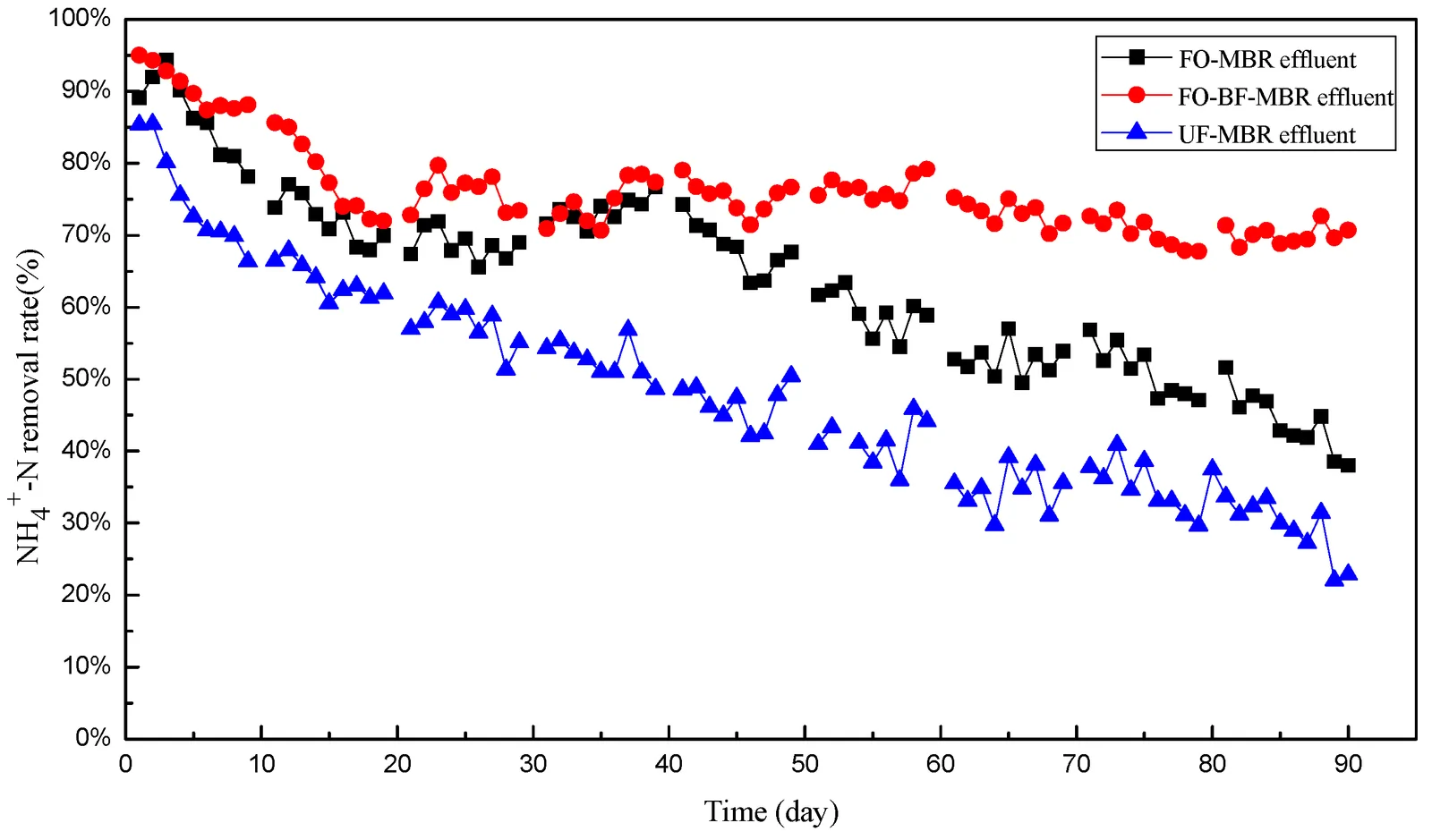
Ammonium nitrogen removal efficiencies of different bioreactors.

The destination and mass balance of nitrogen in MBR mainly contained three pathways: discharge, biodegradation, and microbial assimilation. Hereinto, the conventional TN removal process of biodegradation could be ascribed to the following three stages: (1) Ammonification: This means the process of the decomposition and transformation of nitrogen-containing organic matter into NH_4_^+^ under the metabolism of ammoniated functional bacteria. (2) Nitrification: The nitrification process can be divided into two stages under aerobic conditions. The first stage is the conversion of ammonia nitrogen to nitrite (NO_2_^−^) by nitrosifying, or ammonia-oxidizing bacteria, and the second stage is the conversion of nitrite to nitrate (NO_3_^−^) by nitrifying bacteria. (3) Denitrification: In this process, denitrifying bacteria using nitrate as the electron acceptor while using organic matter (the BOD component in sewage) as the electron donor to provide energy, and can be oxidized to reduce nitrite and nitrate nitrogen to gaseous nitrogen (N_2_) under an anoxic condition. In this study, the retrofit of MBR to OMBR or BF-OMBR used the solo aeration mode and has no anoxic stage; thus, the denitrification process was absent, and the nitrates which came from nitrification stage cannot be converted to N_2_ eventually, and that was why the pre-stage processes such as A^2^/O are arranged as the main procedure of nitrogen removal. Therefore, TN removal in all three of these bioreactors would be impractical [36]. Nevertheless, there are still some interesting discoveries in ammonia, nitrate, and nitrite transforming using AQP FO membranes during the whole span of the experiments (Figure 7).

As described in Figure 7, the removal rate of ammonia nitrogen in these three reactors’ effluent were at the highest level when the experiment started. These results were probably owing to the process that transformed ammonia into nitrate in a thorough nitrification procedure caused by sufficient dissolved oxygen rather than the rejection of the FO membrane. Achilli et al. (2009), Pathak et al. (2018) and Tran et al. (2019) reported this previously [9,32,37]. Due to the lack of a denitrification process, the constant aeration often led to a continuous nitrification process, with the result that the nitrate accumulated in the mixed liquor of the bioreactors were all at a higher level and far exceed the nitrate concentration of the influent [38]. This result also coincides with Holloway et al. (2015) who reported the nitrate concentration was accumulated above 80 mg/L in OMBR without a denitrification process even in an anoxic-OMBR system [15]. For these reason, it seemed nonsensical to talk about the nitrate removal in aerobic MBR under normal temperature and normal C/N ratio conditions whether the FO or UF membranes were in use. However, the temperature was a key factor affecting the biological activity and metabolic ability, and its impact on the nitrification reaction process mainly lay in the growth pattern and biological activity of nitrifying bacteria. The suitable temperature range for aerobic microorganisms was 10~35 °C. When the temperature is below 10 °C or above 30 °C, the growth of nitrifying bacteria significantly slowed down. Therefore, Figure 7 showed the downward trends of the nitrification procedure in the three bioreactors during the whole experiment time under a low-temperature and low-C/N-ratio influent. It also demonstrated the BF-OMBR had the most favorable removal rate of ammonia nitrogen compared to others. These could perhaps be attributed to the fixed biofilm while the other circumstances were almost the same, especially in these two bioreactors. The layered structure of the biofilms could provide a habitat for microorganisms with different ecological characteristics so that it could still maintain high nitrification activity at low temperatures [39]. The double-ring structure of the carrier and the aldehyde fiber bundle constitute a stable spatial structure, which provides an attached surface for the slow-growing nitrifying bacteria, so that they cannot be washed away by the water flow and maintain a long sludge retention time. At low temperature (20 °C to 5 °C), the biomass on the carrier decreased, but still maintained a certain activity, which provided the possibility for the existence of nitrification. Meanwhile, the lowest removal rate of ammonia nitrogen clearly occurred in MBR for the UF membrane which had the unfavorable rejection rate of ammonia nitrogen than the FO membrane under the same experiment conditions.

**Figure 8:**
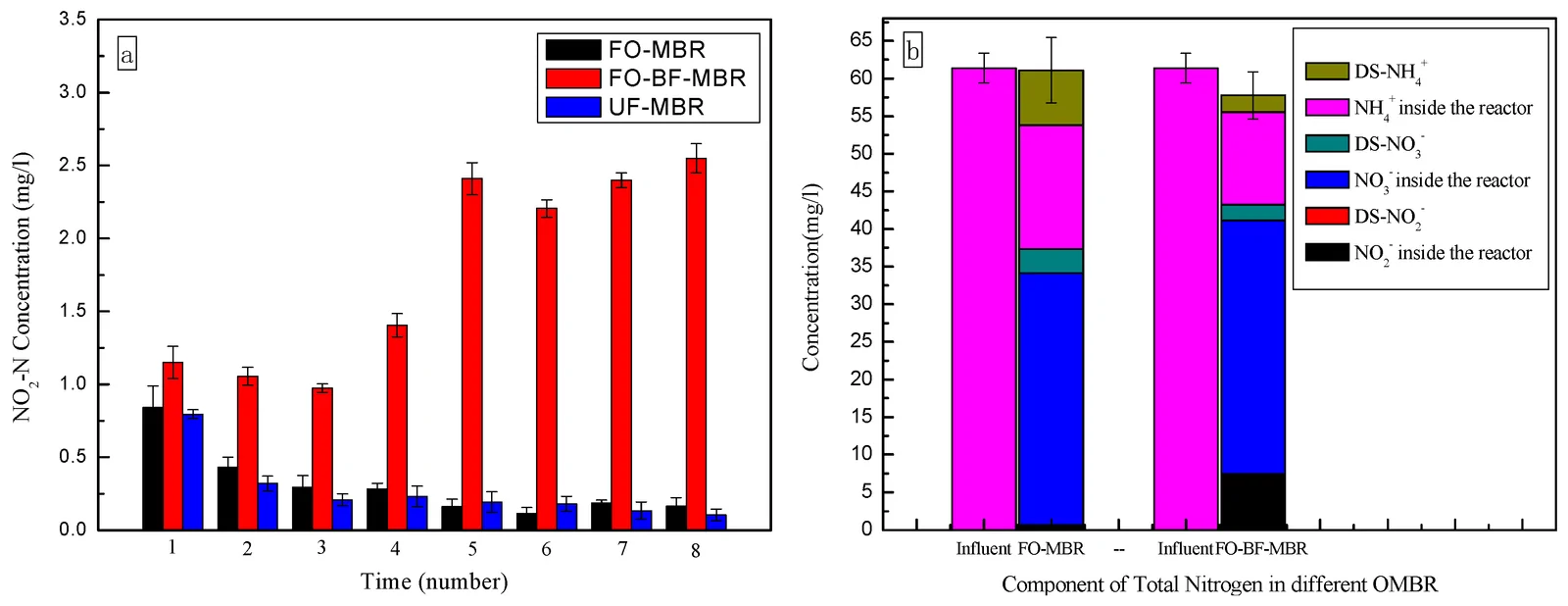
(**a**) Nitrite nitrogen concentrations of different bioreactors; and (**b**) total nitrogen concentration in different reactors under the same influent conditions.

**Figure 9:**
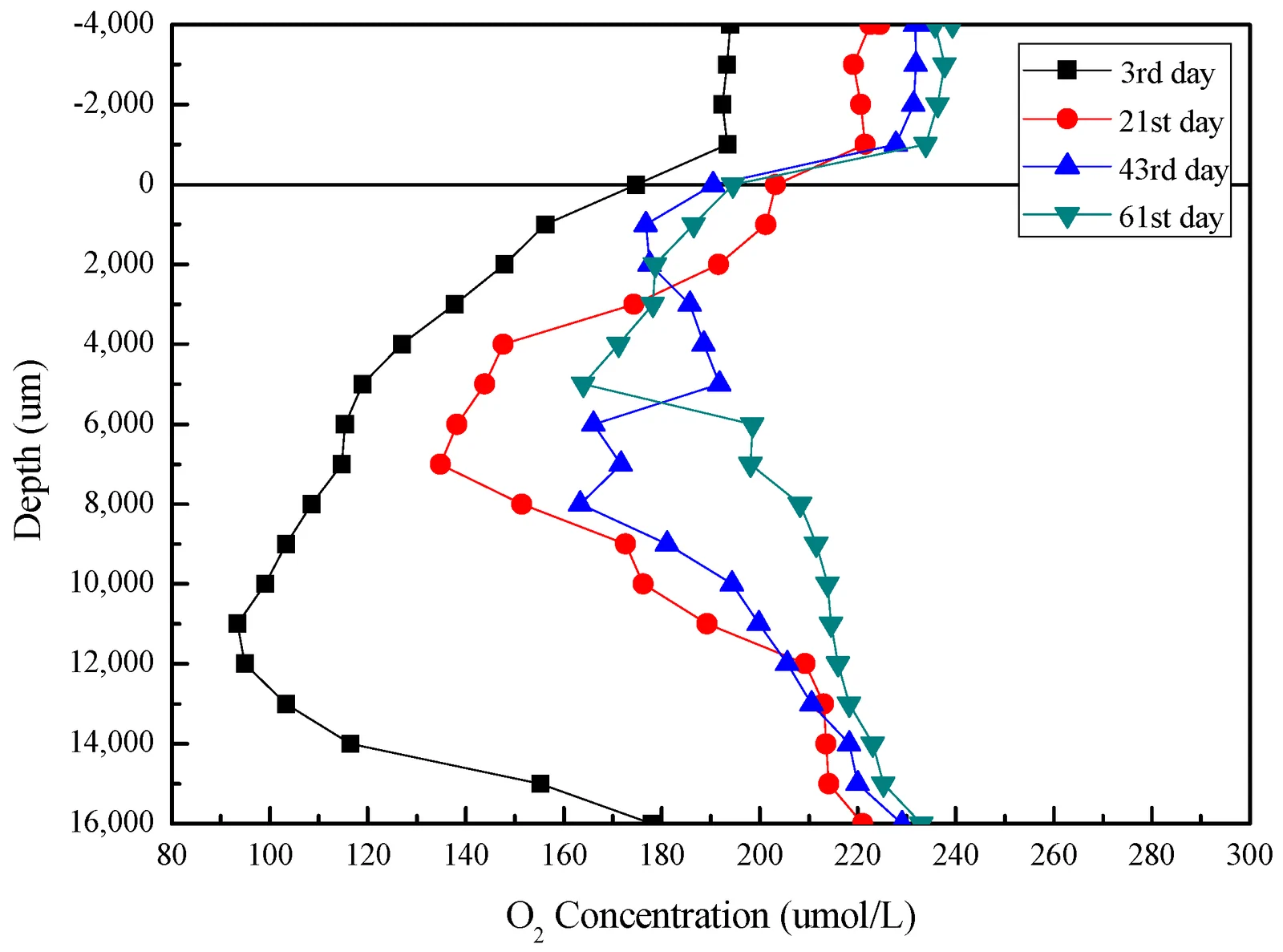
Oxygen concentrations profiles within the biofilm carrier at different depth.

Due to insufficient organic carbon sources, the denitrification process had not been enhanced in all the bioreactors. As could be seen from Figure 8a, the nitrite concentration was at a very low level during the whole experiment time except in BF-OMBR. To some extent, this may suggest the tendency toward some denitrification process in BF-OMBR, and this emergence of denitrification can probably be ascribed to the simultaneous nitrification and denitrification (SND) process by the aid of the biofilm attached in the double-ring filler. SND can achieve the ideal circumstance, relatively, that is to say, carrying out the removal of organic carbon, and the nitrification and denitrification process in one reactor without a need for an additional carbon source, and lower capital investment and operating cost [40,41]. Some researchers reported that the dissolved oxygen level and its gradient in biofilm could form the aerobic and anaerobic zones distributed in the different depths of the biofilm, in which the autotrophic nitrifying and heterotrophic denitrifying microorganisms were coexistent, which makes the SND process possible [42,43]. Special biofilms for SND were easier to form on the surface of the carrier owing to the gradient of the DO concentration and the gradient of the dissolved organic carbon (DOC) from the outer layer to the inner layer; as a result, nitrifying bacteria and denitrifying bacteria were located in the outer layer and inner layer, respectively [44]. The DO distribution in the fixed biofilms of one BF-OMBR carrier at different depths could be seen in Figure 9. The variation curve of the DO concentration in the different inner position of the biofilm under different times showed the evidence of the aerobic and anaerobic zone existence in the initial stage of the experiment. This might imply SND process had a certain possibility in BF-OMBR. The difference between OMBR and BF-OMBR can be seen by comparing the material balance of nitrogen in the two reactors (Figure 8b). Although the total nitrogen reduction in BF-OMBR was very limited, it still indicated the possibility of total nitrogen removal, which could be attributed to the spillover of nitrogen or the greenhouse gas nitrous oxide by SND processes. This may also indicate that the presence of the immobilized biofilm possibly enabled efficient denitrification in the bioreactor even at temperatures as low as 10 °C. Most cases reported in the previous studies suggest the concentration of DO in the bioreactor was controlled at 2–4 mg/L, which can provide an aerobic environment and promote the formation of an anaerobic zone in the biofilm inner space [43,45]. This situation could hardly be generated in the conventional OMBR and UF/MF-MBR due to the fact that the higher level of DO concentration (such as 6~8 mg/L, etc.) in the mixed liquor could strongly restrain the activity of heterotrophic denitrifying microorganisms in the completely mixed activated sludge process. Nevertheless, these SND processes were unable to exist for a long time, which was attributed to the lower C/N ratio (e.g., C/N = 3 or less) of the influent, and this restrained the continued occurrence of SND in the bioreactor mainly due to the carbon source shortage for the heterotrophic denitrification [46,47]. It could also imply the SND process would not easily operate continuously even when fixed-biofilm carriers integrate into the MBR during the effort to retrofit MBR to OMBR simply without any other configuration modification (such as the changing of the aeration mode, etc.) under low-temperature and low-C/N-ratio influents. Tran et al. (2020) reported a baffled OMBR to form an anoxic region to facilitate the denitrification process and to reduce the nitrate to gaseous nitrogen; this may be another way to eliminate the nitrate accumulation in the OMBR except for adjusting the aeration rate to establish the SND process [48].

## 4. Discussion

As the MBR process is often used in rural wastewater treatment applications, there are still some challenges and scope for future study regarding this BF-OMBR. The following recommendations are suggested:

1. Limitations: Firstly, the experiments were conducted using synthetic wastewater to strictly control water quality parameters. While this allows for a fundamental understanding of the process, it may not fully capture the complexity of real rural low-C/N-ratio wastewater, which contains diverse particulate matter, recalcitrant organic compounds, and fluctuating loads that could exacerbate the membrane fouling or alter the biological kinetics. Secondly, the study confirmed the trend of SND through the macroscopic mass balance; due to the limitations in length/conditions, it did not involve microscopic gene validation. In the future, molecular biology methods will be used to further analyze its micro-ecological mechanisms. Finally, although the current experimental design does not allow for the precise decoupling of individual contributions, the observed improvements can be theoretically attributed to the distinct advantages of both components. Based on previous studies, the high intrinsic water permeability of AQP membranes is generally responsible for the enhanced water flux under low-salinity conditions. Meanwhile, the fixed-biofilm carriers likely contributed to better denitrification by providing a protected microenvironment. The integration of these two technologies in the BF-OMBR system appears to leverage these individual benefits. Future studies should include control groups, such as a BF-OMBR equipped with a conventional TFC membrane and an OMBR with an AQP membrane without biofilm carriers, to systematically isolate and evaluate the individual contributions of membrane properties and biofilm effects.
2. Optimize Aeration for TN Removal: Integrating a fixed-biofilm carrier into an osmotic membrane bioreactor (BF-OMBR) can promote the formation of anaerobic zones in the biofilm’s inner space. However, constant aeration is unfavorable for TN removal. An intermittent aeration strategy or another method for DO concentration control should be employed in the reactor to establish a denitrification process and ultimately promote better TN removal.
3. Explore Alternative Fertilizer Draw Solutions: In many rural districts, not only KCl but other fertilizers too are relatively easy to acquire, and their diluents are convenient for agricultural irrigation and hydroponic applications. While the FO process is well-known as a low-energy technology, this recognition is accurate only when DS regeneration is unnecessary. In an actual FDFO process, a mixed solution of simulated compound fertilizer components (e.g., NH_4_NO_3_ + KH_2_PO_4_ or NH_4_H_2_PO_4_ + KCl) should be considered for having the best comprehensive performance (water flux, RSF, and irrigation applicability).
4. Further Research on Retrofitting MBR to OMBR: Retrofitting MBR to OMBR is a possible alternative way to overcome the challenges in rural sewage treatment upgrading, especially under conditions of low-temperature and low-C/N-ratio influents. It can be achieved with little extra operational cost and simple operational training, while providing many advantages. However, the amount of water treated by OMBR is far less than that by MBR, and the experimental data provided so far are still insufficient. More research is needed for the future spreading and use of these integrated/hybrid FO processes.

## 5. Conclusions

In this work, a conventional MBR was retrofitted into an OMBR by replacing the UF membrane with an FO membrane, and a BF-OMBR was further constructed by incorporating fixed-biofilm carriers and an AQP-FO membrane. Although the MBR showed much a higher water flux (10–24 LMH; approximately 3–4 times that of the OMBRs), the data from BF-OMBR suggests it might have achieved better TOC and ammonium nitrogen removal than the MBR and conventional OMBR. Salinity accumulation in the BF-OMBR remained low (<3.5 mS/cm) during 90 d of operation. The improved performance of the BF-OMBR was attributed to the combined effects of membrane rejection and attached-growth biodegradation. However, effective denitrification was not achieved under continuous aeration, and a stable SND process would require an optimized DO control, aeration strategy, and/or carbon supply. Further improvement of the FO membrane’s hydrophilicity, antifouling properties, and cleaning protocols is also needed to enhance the water flux and operational stability. Overall, this study demonstrates the feasibility of using BF-OMBR as a supplementary strategy for maintaining the rural wastewater treatment performance during a winter operation.

## Figures and Tables

**Table 1:** Characteristics of combined double-ring filler.

Item	Technical Data
Size (mm)	Φ150 × 60
Plastic ring diameter (mm)	75
Filler diameter (mm)	150
Distance between slices (mm)	60
Filler density (PCS/m^3^)	About 740
Theoretical specific surface area (m^2^/m^3^)	2600
Unit weigh (Kg/m^3^)	About 4.5
Normal load (KgCOD/m^3^)	3–3.5

**Table 2:** Composition of municipal synthetic wastewater.

Component	Formula	Concentration (mg/L)	TOC (mg/L)	TN (mg/L)	C:N
Glucose	C_6_H_12_O_6_.H_2_O	50	80.74 ± 2.38	61.25 ± 1.97	1:1.2~1.3
Peptone	C_2_H_3_N_3_	50			
Potassium Dihydrogen Phosphate	KH_2_PO_4_	17			
Calcium Chloride	CaCl_2_	17			
Magnesium Sulphate	MgSO_4_.7H_2_O	17			
Ferrous Sulfate	FeSO_4_	10			
Sodium Acetate	CH_3_COONa	112.5			
Urea	CH_4_N_2_O	70			

**Table 3:** Basic information of two FO membranes.

	Water Flux * (L/m^2^.h)	Reverse Salt Flux (g/m^2^.h)	Contact Angle	Rejection to Metal Ions (%)			
				Pb^2+^	Cu^2+^	Ba^2+^	Cr^3+^
TFC-FO	8.8 ± 1	3.7 ± 1	81.7 ± 0.4	99.5 ± 0.1	99.7 ± 0.1	99.4 ± 0.3	99.5 ± 0.1
AQP-FO	14 ± 2	3 ± 1	56.2 ± 0.3	99.9 ± 0.1	99.9 ± 0.1	99.7 ± 0.3	99.6 ± 0.1

* The water flux of these two FO membranes is obtained through experiment, and the FS is DI water and DS is 1M NaCl, AL-FS mode.

**Table 4:** Test conditions of product specifications.

Mode	Active Layer Facing Feed Side (AL-FS)
Feed Solution	Standard Test—Deionized water (4 ppm NaCl, <10 µS/cm Rejection Test—10 ppm of metal ions * in deionized water
Draw Solution	Standard Test—1 mol/L NaCl aqueous solution Rejection Test—1 mol/L glucose solution
Temperature	24~25 °C
Cross-flow Velocity	5.2 cm/s

* (1) Metal ions are Pb^2+^, Cu^2+^, Ba^2+^, and Cr^3+^, respectively, for rejection evaluation.

## Data Availability

The original contributions presented in this study are included in the article. Further inquiries can be directed to the corresponding author.

## References

[1] Deng Y., Wheatley A. (2016). Wastewater Treatment in Chinese Rural Areas. Asian J. Water Environ. Pollut..

[2] Zhang R., Wang Z., Cao Z., Rousseau D., Van Hulle S. (2025). Addressing the rural wastewater treatment dilemma: A techno-environmental-economic analysis. Chem. Eng. J..

[3] Ma G., Xi Z., Chen Y., Xu W., Sun C., Zhuang W., Zhang T., Li D., Pan Y., Li Y.Y. (2024). Biofiltration for low-carbon rural wastewater treatment: A review of advancements and opportunities towards carbon neutrality. J. Environ. Chem. Eng..

[4] Xiao K., Liang S., Wang X., Chen C., Huang X. (2019). Current state and challenges of full-scale membrane bioreactor applications: A critical review. Bioresour. Technol..

[5] Gao T., Wang D., Xia L., Zhao S., Xu R., Zhang H., Meng F., Zhou Z., Chen J., Liu W. (2023). Unveiling the residual membrane foulants in full-scale MBR plant after chemically enhanced backwash: Insights into microbe-associated compounds. Desalination.

[6] Chekli L., Phuntsho S., Kim J.E., Kim J., Choi J.Y., Choi J.-S., Kim S., Kim J.H., Hong S., Sohn J. (2016). A comprehensive review of hybrid forward osmosis systems: Performance, applications and future prospects. J. Membr. Sci..

[7] Singh N., Petrinic I., Hélix-Nielsen C., Basu S., Balakrishnan M. (2019). Influence of Forward Osmosis (FO) membrane properties on dewatering of molasses distillery wastewater. J. Water Process Eng..

[8] Zhang X., Liu Y. (2022). Circular economy is game-changing municipal wastewater treatment technology towards energy and carbon neutrality. Chem. Eng. J..

[9] Achilli A., Cath T.Y., Marchand E.A., Childress A.E. (2009). The forward osmosis membrane bioreactor: A low fouling alternative to MBR processes. Desalination.

[10] Wang X., Chang V.W.C., Tang C.Y. (2016). Osmotic membrane bioreactor (OMBR) technology for wastewater treatment and reclamation: Advances, challenges, and prospects for the future. J. Membr. Sci..

[11] Li S., Kim Y., Chekli L., Phuntsho S., Shon H.K., Leiknes T.O., Ghaffour N. (2017). Impact of reverse nutrient diffusion on membrane biofouling in fertilizer-drawn forward osmosis. J. Membr. Sci..

[12] Lambrechts R., Sheldon M.S. (2019). Performance and energy consumption evaluation of a fertiliser drawn forward osmosis (FDFO) system for water recovery from brackish water. Desalination.

[13] Blandin G., Gautier C., Sauchelli Toran M., Monclús H., Rodriguez-Roda I., Comas J. (2018). Retrofitting membrane bioreactor (MBR) into osmotic membrane bioreactor (OMBR): A pilot scale study. Chem. Eng. J..

[14] Geng Y., Yang L., Lian C.A., Pavlostathis S.G., Qiu Z., Xiong Z., Liu Y., Li B., Hu J., Fan W. (2024). Resourceful application and mechanism of oyster shell-microalgae synergistic system: Sustainable treatment of harsh low carbon nitrogen ratio actual wastewater. Environ. Res..

[15] Holloway R.W., Wait A.S., Fernandes da Silva A., Herron J., Schutter M.D., Lampi K., Cath T.Y. (2015). Long-term pilot scale investigation of novel hybrid ultrafiltration-osmotic membrane bioreactors. Desalination.

[16] Gurung K., Ncibi M.C., Sillanpää M. (2017). Assessing membrane fouling and the performance of pilot-scale membrane bioreactor (MBR) to treat real municipal wastewater during winter season in Nordic regions. Sci. Total Environ..

[17] Drews A. (2010). Membrane fouling in membrane bioreactors-characterization, contradictions, cause and cures. J. Membr. Sci..

[18] Wang L., Yu X., Xiong W., Li P., Wang S., Fan A., Su H. (2020). Enhancing robustness of aerobic granule sludge under low C/N ratios with addition of kitchen wastewater. J. Environ. Manag..

[19] Al Aani S., Mustafa T.N., Hilal N. (2020). Ultrafiltration membranes for wastewater and water process engineering: A comprehensive statistical review over the past decade. J. Water Process Eng..

[20] Praveen P., Loh K.C. (2016). Nitrogen and phosphorus removal from tertiary wastewater in an osmotic membrane photobioreactor. Bioresour. Technol..

[21] Yogarathinam L.T., Ismail A.F., Goh P.S. (2024). Membrane Bioreactor for Sewage Treatment. Clean Water: Next Generation Technologies.

[22] Jang D., Chung Y., Jang A., Kang S. (2022). Role of organic fouling layers on the transport of micropollutants in forward osmosis membrane processes. J. Water Process Eng..

[23] Zhai S.Q., Nan J., Cai W.J., He X.W., Yang X.L., Yang Y.L., Song H.L. (2025). Interaction mechanism between membrane biofouling and reverse solute diffusion in an osmotic membrane bioreactor. J. Water Process Eng..

[24] Song W., You H., Li Z., Liu F., Qi P., Wang F., Li Y. (2017). Membrane fouling mitigation in a moving bed membrane bioreactor combined with anoxic biofilter for treatment of saline wastewater from mariculture. Bioresour. Technol..

[25] Zhu W., Wang X., She Q., Li X., Ren Y. (2018). Osmotic membrane bioreactors assisted with microfiltration membrane for salinity control (MF-OMBR) operating at high sludge concentrations: Performance and implications. Chem. Eng. J..

[26] Li S., Duan L., Zhang H., Zhao Y., Li M., Jia Y., Gao Q., Yu H. (2024). Critical review on salt tolerance improvement and salt accumulation inhibition strategies of osmotic membrane bioreactors. Bioresour. Technol..

[27] Sun C., Leiknes T., Weitzenböck J., Thorstensen B. (2010). Salinity effect on a biofilm-MBR process for shipboard wastewater treatment. Sep. Purif. Technol..

[28] Fuwad A., Ryu H., Han E.D., Lee J.H., Malmstadt N., Kim Y.R., Seo Y.H., Kim S.M., Jeon T.J. (2024). Highly permeable and shelf-stable aquaporin biomimetic membrane based on an anodic aluminum oxide substrate. npj Clean Water.

[29] Sridang P.C., Pottier A., Wisniewski C., Grasmick A. (2008). Performance and microbial surveying in submerged membrane bioreactor for seafood processing wastewater treatment. J. Membr. Sci..

[30] Jørgensen M.K., Sørensen J.H., Quist-Jensen C.A., Christensen M.L. (2018). Wastewater treatment and concentration of phosphorus with the hybrid osmotic microfiltration bioreactor. J. Membr. Sci..

[31] Luján-Facundo M.J., Soler-Cabezas J.L., Mendoza-Roca J.A., Vincent-Vela M.C., Bes-Piá A., Doñate-Hernández S. (2017). A study of the osmotic membrane bioreactor process using a sodium chloride solution and an industrial effluent as draw solutions. Chem. Eng. J..

[32] Pathak N., Li S., Kim Y., Chekli L., Phuntsho S., Jang A., Ghaffour N., Leiknes T., Shon H.K. (2018). Assessing the removal of organic micropollutants by a novel baffled osmotic membrane bioreactor-microfiltration hybrid system. Bioresour. Technol..

[33] Rout V.K., Setia G., Kaur S., Kumar L., Kumar S. (2024). Membrane Bioreactors. Advancements in Bio-systems and Technologies for Wastewater Treatment.

[34] Kumar M., Lee P.-Y., Fukusihma T., Whang L.-M., Lin J.-G. (2012). Effect of supplementary carbon addition in the treatment of low C/N high-technology industrial wastewater by MBR. Bioresour. Technol..

[35] Huang C., Shi Y., Xue J., Zhang Y., El-Din M.G., Liu Y. (2017). Comparison of biomass from integrated fixed-film activated sludge (IFAS), moving bed biofilm reactor (MBBR) and membrane bioreactor (MBR) treating recalcitrant organics: Importance of attached biomass. J. Hazard. Mater..

[36] Xia Z., Wang Q., She Z., Gao M., Zhao Y., Guo L., Jin C. (2019). Nitrogen removal pathway and dynamics of microbial community with the increase of salinity in simultaneous nitrification and denitrification process. Sci. Total Environ..

[37] Tran V.H., Lim S., Han D.S., Pathak N., Akther N., Phuntsho S., Park H., Shon H.K. (2019). Efficient fouling control using outer-selective hollow fiber thin-film composite membranes for osmotic membrane bioreactor applications. Bioresour. Technol..

[38] Qiu G., Ting Y.P. (2013). Osmotic membrane bioreactor for wastewater treatment and the effect of salt accumulation on system performance and microbial community dynamics. Bioresour. Technol..

[39] Soliman M., Eldyasti A. (2018). Ammonia-oxidizing bacteria (AOB): Opportunities and applications—A review. Rev. Environ. Sci. Bio/Technol..

[40] Hibiya K., Terada A., Tsuneda S., Hirata A. (2003). Simultaneous nitrification and denitrification by controlling vertical and horizontal microenvironment in a membrane-aerated biofilm reactor. J. Biotechnol..

[41] Pathak N., Phuntsho S., Tran V.H., Johir M.A.H., Ghaffour N., Leiknes T., Fujioka T., Shon H.K. (2020). Simultaneous nitrification-denitrification using baffled osmotic membrane bioreactor-microfiltration hybrid system at different oxic-anoxic conditions for wastewater treatment. J. Environ. Manag..

[42] Chen H., Liu S., Yang F., Xue Y., Wang T. (2009). The development of simultaneous partial nitrification, ANAMMOX and denitrification (SNAD) process in a single reactor for nitrogen removal. Bioresour. Technol..

[43] Song Z., Zhang X., Ngo H.H., Guo W., Song P., Zhang Y., Wen H., Guo J. (2019). Zeolite powder based polyurethane sponges as biocarriers in moving bed biofilm reactor for improving nitrogen removal of municipal wastewater. Sci. Total Environ..

[44] Wu Y., Song H.L., Pan Y., Zhai S.Q., Shao Y., Nan J., Yang Y.L., Zhang L.M. (2022). Insight into the role of microbial community interactions on nitrogen removal facilitated by a bioelectrochemical system in an osmotic membrane bioreactor. Bioresour. Technol..

[45] Li T., Yang X.L., Song H.L., Wu J.J., Xu J.Y. (2019). Alkali-treated cellulose carrier enhancing denitrification in membrane bioreactor. Int. Biodeterior. Biodegrad..

[46] Matsumoto S., Terada A., Tsuneda S. (2007). Modeling of membrane-aerated biofilm: Effects of C/N ratio, biofilm thickness and surface loading of oxygen on feasibility of simultaneous nitrification and denitrification. Biochem. Eng. J..

[47] Machat H., Boudokhane C., Roche N., Dhaouadi H. (2019). Effects of C/N Ratio and DO concentration on Carbon and Nitrogen removals in a Hybrid Biological Reactor. Biochem. Eng. J..

[48] Tran V.H., Pathak N., Shon H.Y. (2020). Osmotic membrane bioreactor technology-Concept, potential and challenges. Current Developments in Biotechnology and Bioengineering.

